# Electronic Medical Record Inaccuracies: Multicenter Analysis of Challenges with Modified Lung Cancer Screening Criteria

**DOI:** 10.1155/2020/7142568

**Published:** 2020-03-26

**Authors:** Candice L. Wilshire, Carson C. Fuller, Christopher R. Gilbert, John R. Handy, Kimberly E. Costas, Brian E. Louie, Ralph W. Aye, Alexander S. Farivar, Eric Vallières, Jed A. Gorden

**Affiliations:** ^1^Division of Interventional Pulmonology and Thoracic Surgery, Swedish Cancer Institute, Seattle, WA, USA; ^2^Department of Thoracic Surgery, Providence Health and Services, Portland, OR, USA; ^3^Department of Thoracic Surgery, Providence Medical Group, Everett, WA, USA

## Abstract

The National Comprehensive Cancer Network expanded their lung cancer screening (LCS) criteria to comprise one additional clinical risk factor, including chronic obstructive pulmonary disease (COPD). The electronic medical record (EMR) is a source of clinical information that could identify high-risk populations for LCS, including a diagnosis of COPD; however, an unsubstantiated COPD diagnosis in the EMR may lead to inappropriate LCS referrals. We aimed to detect the prevalence of unsubstantiated COPD diagnosis in the EMR for LCS referrals, to determine the efficacy of utilizing the EMR as an accurate population-based eligibility screening “trigger” using modified clinical criteria. We performed a multicenter review of all individuals referred to three LCS programs from 2012 to 2015. Each individual's EMR was searched for COPD diagnostic terms and the presence of a diagnostic pulmonary functionality test (PFT). An unsubstantiated COPD diagnosis was defined by an individual's EMR containing a COPD term with no PFTs present, or the presence of PFTs without evidence of obstruction. A total of 2834 referred individuals were identified, of which 30% (840/2834) had a COPD term present in their EMR. Of these, 68% (571/840) were considered unsubstantiated diagnoses: 86% (489/571) due to absent PFTs and 14% (82/571) due to PFTs demonstrating no evidence of postbronchodilation obstruction. A large proportion of individuals referred for LCS may have an unsubstantiated COPD diagnosis within their EMR. Thus, utilizing the EMR as a population-based eligibility screening tool, employing expanded criteria, may lead to individuals being referred, potentially, inappropriately for LCS.

## 1. Introduction

As lung cancer screening programs (LCSPs) proliferate outside the research environment, debate regarding the optimal population for screening continues. Fodder for this debate comes from advocates and societies which recommend expanding lung cancer screening (LCS) criteria to individuals with a lung cancer risk based on clinical factors other than smoking and age [1, 2]. While medical society guidelines hue close to the Center for Medicare and Medicaid Services inclusion criteria, the National Comprehensive Cancer Network (NCCN) and the American Association for Thoracic Surgery (AATS) recommend modified risk criteria. These criteria allow for a decrease in both age and tobacco pack year when a diagnosis of chronic lung disease, including chronic obstructive pulmonary disease (COPD), is present. COPD is an independent risk factor for lung cancer, but it is often misclassified and unsubstantiated in the medical record [3–8].

The electronic medical record (EMR) is used as a population-based screening tool by hospitals and healthcare networks for identification of at-risk populations, including lung cancer screening. These EMR “triggers” or best practice alerts (BPA) draw from predetermined, autopopulated EMR fields, which are dependent on the quality of the entered data. Inaccurate data used to trigger the BPA and prompt LCS could result in inappropriate populations being screened, potentially impacting the balance of benefits versus harms as LCSPs proliferate.

We aimed to detect the prevalence of unsubstantiated COPD diagnosis in the EMR of individuals referred for LCS, to determine the efficacy of utilizing the EMR as an accurate population-based eligibility screening “trigger” using modified clinical criteria.

## 2. Materials and Methods

### 2.1. Study Population

We reviewed individuals who were referred to three separate LCSPs between January 1, 2012, and February 29, 2016. The LCSPs included were those based on the Swedish Cancer Institute in Seattle, Washington State; Providence Regional Cancer Partnership in Everett, Washington State; and Providence Health and Services in Portland, Oregon. Data from all individuals referred to the three LCSPs were pooled and is presented as such. The Swedish Cancer Institute Institutional Review Board approved this study (SWD5896S-15) and provided coverage for the other affiliated sites, and the requirement for informed consent was waived.

### 2.2. Electronic Medical Record Review

Each individual's EMR (EPIC Hyperspace Version 6.0, Verona, Wisconsin) was searched for the obstructive pulmonary disease terms “COPD and chronic airway obstruction,” as well as “chronic bronchitis and emphysema” as these terms are often interchanged with COPD terms. The first location where the term was identified in the EMR, as well as the specialty/title of the individual who entered the term, was captured. To determine if the presence of the term in the individual's EMR as a diagnosis was substantiated, we searched for the presence of a confirmatory pulmonary function test (PFT).

### 2.3. Substantiated versus Unsubstantiated Diagnosis

A substantiated COPD diagnosis was defined by the presence of a confirmatory PFT as per the Global Initiative for Chronic Obstructive Lung Disease (GOLD) criteria: a postbronchodilator FEV_1_/FVC < 0.70 [9].

A diagnosis was considered unsubstantiated when an obstructive term was present in an individual's EMR with either no PFTs present (even if mentioned as previously performed), or when PFTs were present that were negative or without evidence of sustained postbronchodilation obstruction.

### 2.4. Statistical Analysis

Data are reported as counts and percentages, as well as median and 25^th^–75^th^ interquartile ranges. Data summary was performed in Microsoft Excel (Microsoft Office 2013; Redmond, Washington).

## 3. Results

We identified a total of 2834 referred individuals, of which the median age was 63 years (IQR: 59–68) and 53% (1510) were male.

### 3.1. Obstructive Terms Present, Unsubstantiated Diagnosis

**Figure 1:**
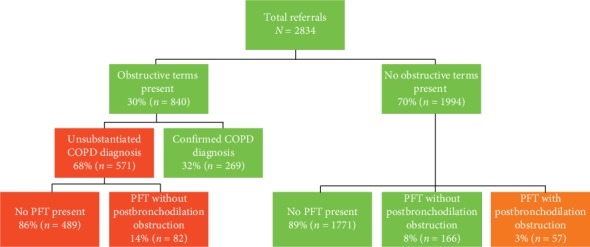
Flow diagram depicting lung cancer screening program referrals stratified by the presence or absence of pulmonary airway obstructive terms in the individual's electronic medical record and whether these were substantiated.

An obstructive pulmonary disease term was present in 30% (840/2834) of referred individual's EMRs (Figure 1). Of these, 68% (571/840) were considered unsubstantiated diagnoses: 86% (489/571) due to absence of a PFT and 14% (82/571) due to a PFT demonstrating no evidence of postbronchodilation obstruction. Thus, 20% (571/2834) of the total referred population had an unsubstantiated diagnosis.

Ninety-one percent (521/571) of individuals had a diagnosis with at least the term “obstructive,” while 4% (20/571) and 5% (30/571) had the isolated terms “chronic bronchitis” and “emphysema,” respectively.

The most prominent locations in the EMR where the unsubstantiated term was first identified was the “problem list” (83%, 473/571) and the “history” tab (16%, 89/571). Only a minority had terms identified within notes in the chart (1%, 9/571).

**Figure 2:**
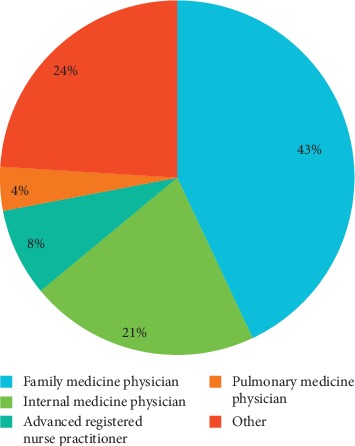
Specialty/title of the individuals who entered an unsubstantiated pulmonary airway obstructive term.

The most frequent specialty of individuals entering an unsubstantiated term in the EMR was family medicine (Figure 2).

### 3.2. Obstructive Terms Present, Substantiated Diagnosis

Of those with obstructive terms present, 32% (269/840) had confirmation of their COPD diagnosis with positive PFTs present in the EMR (Figure 1).

### 3.3. No Obstructive Terms Present

Of those referrals with no obstructive terms present in their EMR (70%, 1994/2834), the majority had either no PFTs present (89%, 1771/1994) or a negative PFT (8%, 166/1994), while 3% (57/1994) had PFTs diagnostic of COPD (Figure 1).

## 4. Comment

This is one of the first studies to address the challenges posed by EMR-identified high-risk individuals for LCS using modified clinical criteria in a large population of screened individuals from multiple centers. We identified that 20% of all individuals referred for LCS had an unsubstantiated diagnosis of obstructive airway disease within their EMR. The integrity of clinical data on COPD within the EMR poses a potential challenge to identification of an alternative risk population eligible for LCS based on the clinical diagnosis of COPD as proposed by the NCCN expanded criteria.

Although it is often quoted that the number needed to screen to prevent one lung cancer death is 320, further analysis of the NLST demonstrated that screening risk is more nuanced and there is variable risk within that population [8, 10]. The results of the analysis confirmed that tailoring of low-dose CT screening to a patient's predicted risk of lung cancer death could narrow the eligible population without loss of the benefits of screening or a disproportionate increase in harms [10]. By restricting screening to the 60% of participants at the highest risk for death from lung cancer within five years (the three highest quintiles of risk of the five), as compared with the entire group, 88% of preventable lung cancer deaths were captured, the number of participants needed to be screened to prevent one lung cancer death was reduced from 302 to 161, and the number of false positive results per prevented lung cancer death was reduced from 108 to 65. Efforts to better define individuals and populations at risk are active areas of study. Not all screening trials have demonstrated a survival benefit as seen in the NLST. Both the Detection And screening of early lung cancer with Novel imaging Technology (DANTE) trial and the Danish Lung Cancer Screening Trial (DLCST) failed to show a lung cancer survival advantage through screening [11, 12]. One possible explanation for this is reduced eligibility criteria of age (60–74 years for the DANTE trial and 50–70 years for the DLCST trial) and tobacco exposure (minimum of 20 pack-years of smoking), reducing the screened populations risk and potentially missing the benefit of screening [13, 14].

The NCCN and AATS have published modified criteria for LCS in individuals with pulmonary disease, specifically pulmonary fibrosis and COPD, and while smoking is the leading risk factor for the development of lung cancer, it is also the leading contributor to COPD, so it is logical that overlap between the two exists [6, 7]. Studies suggest that COPD affects nearly a quarter of smokers and is reported in 40–80% of smoking-related lung cancer patients [15–18]. Historically, studies reported on the intimate relationship between the incidence and clinical course of lung cancer and COPD, suggesting COPD may only be a risk factor for lung cancer; however, more recently, many cohort studies, including LCS trials, have demonstrated a 2–4 times greater risk of incident lung cancer in individuals with COPD/emphysema compared to those without [5, 19–21]. In addition, a recent study suggested a linear relationship between the severity of COPD and incidence of lung cancer, with the risk of lung cancer correlating to increasing GOLD classification, independent of cigarette smoking status [22].

Our data demonstrate that 20% of our population did not have a substantiated diagnosis of COPD. The misdiagnosis of obstructive lung disease is not a new one, and other studies of both COPD and asthma have demonstrated misdiagnosis and/or lack of substantiated diagnosis [16, 23–26]. The diagnosis of COPD remains based on the documentation of irreversible airflow obstruction, developed to determine disease severity, as well as to help avoid misdiagnosis [9]. A recent Veterans Affairs health system study of hospitalized patients demonstrated that 21% had no spirometric measurements and 11% had normal pre- or postbronchodilator measurements, despite a discharge diagnosis of COPD [27]. In addition, a Canadian report from three primary care sites demonstrated that 21% of patients over 40 years with a minimum smoking history of 20 pack-years had spirometry consistent with COPD, but only 30% of these individuals had been correctly diagnosed with COPD prior to the study [28]. Similarly, other studies have reported variable rates of spirometry confirming COPD that range from 9 to 70% [23–26]. In addition, the prevalence of COPD in the US surveillance data from 1999 to 2011 was based in-part on the Behavioral Risk Factor Surveillance System and in-part on the National Health Interview Survey [29]. In both these surveys, the definition of COPD was based on responses to the survey questions: have you ever been told by a doctor or other health professional that you have COPD, emphysema, or chronic bronchitis?

It is well established that LCS of high-risk individuals with low-dose CT demonstrates a mortality benefit [8]. However, despite recommendations, only a relatively small proportion of eligible individuals are currently being screened, as evidenced by low numbers of program enrollees relative to the estimated 7 million individuals in the US who would meet eligibility criteria [8, 30]. While multiple interventions will be required to target this challenge, a solution currently being explored is the mass identification of high-risk individuals through population-based eligibility screening utilizing the EMR [31]. The EMR is the principle source of relevant clinical information and is increasingly used for management of chronic disease such as COPD [32, 33].

While this concept of identifying eligible individuals with a population-based screening tool, such as an EMR search, is attractive; in reality, this should be approached with caution as data reliability has been shown to be unpredictable. We previously demonstrated that data entered into the EMR is not necessarily accurate, as we identified that there was a 96% discordance rate in the pack-year smoking history obtained from the EMR versus the shared decision-making conversation at the time of LCS [34]. Other studies have reported similar concerns. A recent study identified that the sensitivity of the problem list for identifying common major comorbidities, including COPD, was poor and ranged from 1 to 46% [35]. In addition, a report of EMR triage medication history recorded on emergency department visits identified accuracy in only 22% of cases, and a separate report assessing EMRs accompanying referral requests by physicians for plastic surgery consultation identified frequent inaccuracies and incomplete data fields [36, 37].

There are several limitations to this study. First, the retrospective study design is associated with an inherent bias. Second, we did not attempt to investigate if PFTs were performed at an outside institution if the diagnosis of COPD was made prior to the individual coming into contact with our system. However, missing this information was likely minimized as previous notes should be scanned into the EMR on referral. Lastly, geographic clustering of COPD prevalence, medicare hospitalizations, and COPD-related mortality has been reported, particularly along the Ohio River Valley and several Western and Southern States [29]. This regional variation in COPD burden could limit the generalizability of our results.

In conclusion, this study demonstrates that a large proportion of individuals referred to LCSPs may have an unsubstantiated diagnosis of COPD within their EMR. Utilization of the EMR as a population-based eligibility screening tool may lead to a significant proportion of individuals being inappropriately referred for LCS. Utilization of expanded criteria, adding the clinical diagnosis of COPD and lowering the eligibility age and tobacco pack-year, to broaden the risk pool may be challenged by a high rate of unsubstantiated diagnosis. Careful review of charts and accurate documentation, as well as employment of more objective metrics to refine the high-risk populations for LCS referral, is needed.

## Data Availability

The data used to support the findings of this study are available from the corresponding author upon request.

## Abbreviation

AATS: American Association for Thoracic Surgery
CMS: Centers for Medicare and Medicaid Services
COPD: Chronic obstructive pulmonary disease
CT: Computed tomography
DANTE: Detection And screening of early lung cancer with Novel imaging Technology
DLCST: Danish Lung Cancer Screening Trial
EMR: Electronic medical record
FEV_1_: Forced expiratory volume in one second
FVC: Forced vital capacity
GOLD: Global Initiative for Chronic Obstructive Lung Disease
LCS: Lung cancer screening
LCSP: Lung cancer screening program
NCCN: National Comprehensive Cancer Network
NLST: National Lung Screening Trial
PFT: Pulmonary function test
US: United States.
